# A network-patch methodology for adapting agent-based models for directly transmitted disease to mosquito-borne disease

**DOI:** 10.1080/17513758.2015.1005698

**Published:** 2015

**Authors:** Carrie A. Manore, Kyle S. Hickmann, James M. Hyman, Ivo M. Foppa, Justin K. Davis, Dawn M. Wesson, Christopher N. Mores

**Affiliations:** a Center for Computational Science, Department of Mathematics, Tulane University, New Orleans, LA 70118, USA; b Battelle/Epidemiology & Prevention Branch, Influenza Division, CDC, Atlanta, GA, USA; c Department of Tropical Medicine, School of Public Health and Tropical Medicine, Tulane University, New Orleans, LA 70118, USA; d Vector-borne Disease Laboratories, Center for Experimental Infectious Disease Research, Department of Pathobiological Sciences, School of Veterinary Medicine, Louisiana State University, Baton Rouge, LA 70803, USA

**Keywords:** patch, network, dengue, chikungunya, mosquito-borne disease, differential equationsmodel, individual-based model

## Abstract

Mosquito-borne diseases cause significant public health burden and are widely re-emerging or emerging. Understanding, predicting, and mitigating the spread of mosquito-borne disease in diverse populations and geographies are ongoing modelling challenges. We propose a hybrid network-patch model for the spread of mosquito-borne pathogens that accounts for individual movement through mosquito habitats, extending the capabilities of existing agent-based models (ABMs) to include vector-borne diseases. The ABM are coupled with differential equations representing ‘clouds’ of mosquitoes in patches accounting for mosquito ecology. We adapted an ABM for humans using this method and investigated the importance of heterogeneity in pathogen spread, motivating the utility of models of individual behaviour. We observed that the final epidemic size is greater in patch models with a high risk patch frequently visited than in a homogeneous model. Our hybrid model quantifies the importance of the heterogeneity in the spread of mosquito-borne pathogens, guiding mitigation strategies.

## 1. Introduction

Predicting the spread of mosquito-borne pathogens, such as dengue virus, chikungunya virus, Rift Valley fever, and West Nile virus is complicated by complexity of the systems, lack of appropriately granular data, and computational expense of realistic models. The existing models for the spatial spread of mosquito-borne pathogens, while providing important insight into disease dynamics, often ignore either detailed host movement and/or explicit mosquito population dynamics to reduce complexity and computational expense [1,4,33,39]. These and other studies have shown that capturing host behaviour and movement through the mosquito environment is important and perhaps even crucial to understanding risk and informing mitigation efforts [1,32,34,36]. We bridge this gap by combining an agent-based spatial model (ABM) for host movement on a network with a patch-based ordinary differential equation (ODE) model that captures environmental and mosquito dynamics in geographic habitat patches that cover the region modelled by the ABM. In particular, we introduce a relatively simple methodology for extending already existing large-scale ABMs for hosts to include mosquito-borne disease. This ‘network-patch’ model will help quantify the importance of heterogeneity in these components and aid in predicting the spread of mosquito-borne pathogens, particularly in forecasting the initial spread following introduction into a new location. The hybrid model described here focuses on the transmission dynamics of mosquito-borne pathogens, but extensions are possible to other arthropod vectors and zoonotic or animal vector-borne pathogens.

The network-patch methodology can be used to adapt existing ABMs for person-to-person transmitted diseases, such as influenza (e.g. EpiSimS [17,30], Framework for Reconstructing Epidemiological Dynamics (FRED) [19], DISimS [8]), to mosquito-borne pathogens. Similarly, ABMs for animal movement could be adapted to include mosquito-borne pathogen spread in addition to directly transmitted pathogens. The method accounts for explicit spatial arrangement of mosquito habitat, the social aspects of host behaviour, and variations in environment and weather. Adams and Kapan [1] modelled spatial mosquito-borne disease on a network where each network node corresponded to exactly one patch and where the mosquito populations did not explicitly depend on weather or landscape. We expand and extend their model for mosquito patches that incorporate variation in mosquito density, determined by biotic (e.g. vegetation, predators) and abiotic factors (e.g. temperature, humidity, and breeding sites) [4,39]. Perkins *et al.* [32] explored the idea of different habitat patches for various mosquito life cycle stages (blood seeking, resting, and oviposition) with aggregated movement of humans between patches based on proportion of time spent in each of the patches. We extend this idea by coupling mosquito habitat patches with ABMs that have already been tuned to model human behaviour and movement in a city or region.

There are several ABMs for mosquito-borne disease where mosquitoes are treated as independent agents [3,9,14,16,31]. The results of such models highlight the role of heterogeneity in host movement, mosquito distribution and density, and the environment in mosquito-borne disease spread. However, they are restricted in spatial scale by the computational cost of modelling each individual mosquito and host. Since relatively little data are available for individual mosquito host-seeking behaviour across larger scales, there is significant parametric uncertainty associated with models for individual mosquitoes, particularly on a large scale. Our approach can easily incorporate larger host populations and a wider geographical area. We account for heterogeneity in disease spread on the spatial scale of patches, rather than the exact spatial location of the individual mosquitoes, as in the related research described in [5,22,25,28,41]. Our model presents a unified framework useful for simulating emerging epidemics, and understanding the roles of spatial, ecological, environmental, social, and behavioural heterogeneities in mosquito-borne diseases.

**Figure 1:**
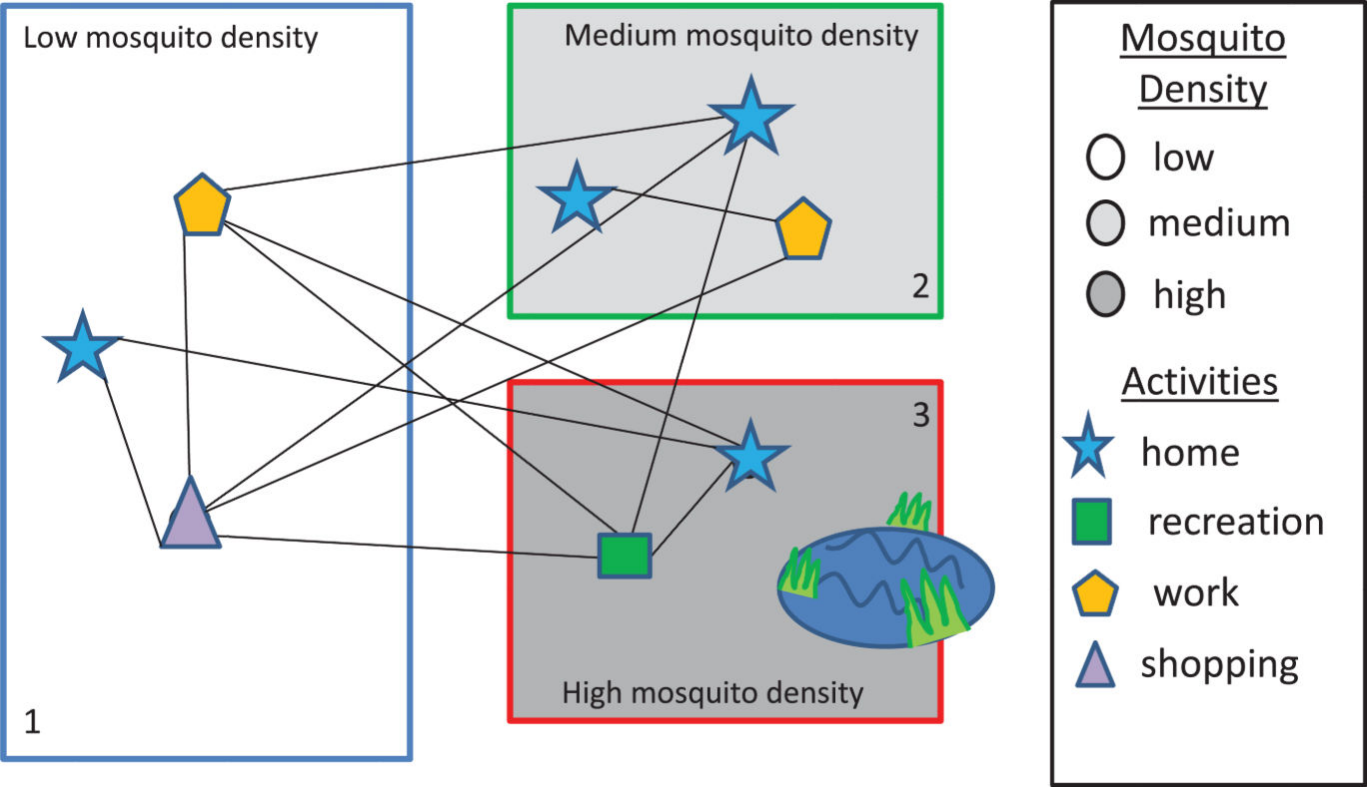
The network-patch model combines the detailed host movement captured by an agent-based spatial network model with a habitat patch model for mosquitoes. The agents in the network model move between locations and activities (network nodes) determined by population, demographics, and host behaviour. We give examples of human activities here. Animal activities could include foraging, drinking, and sleeping locations. Each node is associated with an environmental patch where the local population of infected and uninfected mosquitoes determine the risk of an individual becoming infected while in the patch.

To create the network-patch model, we overlay geographic patches based on the environmental properties correlated with the mosquito's life cycle on a connected host network. The location and size of the mosquito patches is determined by landscape, vegetation, weather, human socioeconomic factors, land use, and availability of mosquito breeding sites. The level of detail used in modelling the mosquito patches will depend on available data and expert opinion. Many of the existing ABMs have the agents located at nodes based on location (e.g. office building, school, and home). For the network-patch model, these location nodes are assigned to a specific patch, and the activity of the individual is then mapped to the appropriate activity category in the patch. Each activity category within a patch is assigned a relative risk of being bitten by a mosquito. Figure 1 illustrates how each patch can encompass several network nodes and edges so that any individual at or between nodes is exposed to the hazards in the patch.

**Figure 2:**
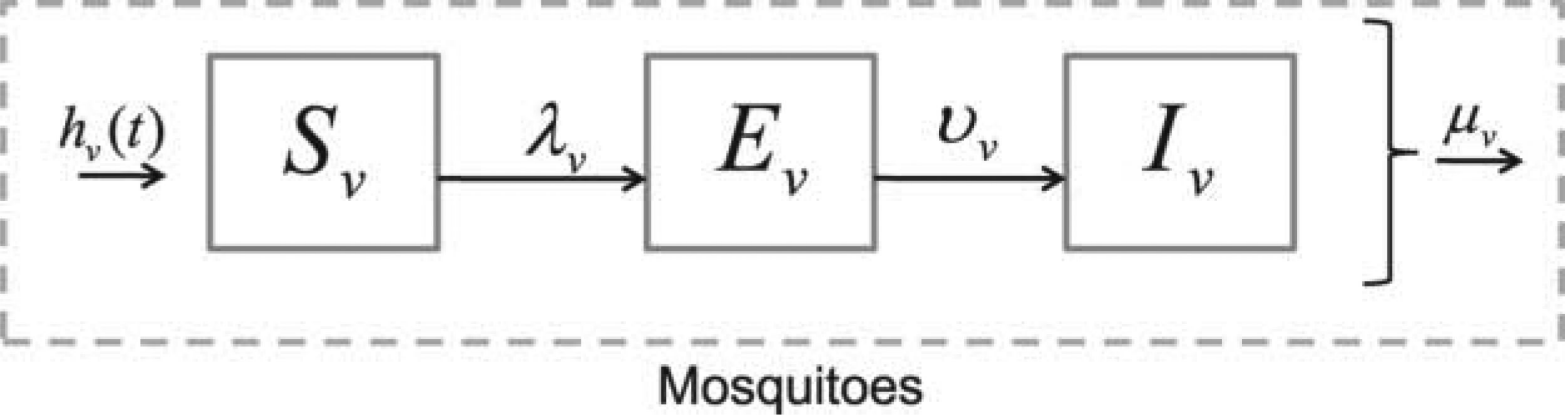
In the female mosquito model, susceptible adults are infected at a rate *λ_v_* and pass through the exposed compartment, *E_v_*, to the infectious compartment, *I_v_*. All compartments contribute to reproduction, and we assume the death rate is independent of the infection status.

The mosquito dynamics in each patch are simulated using systems of ODEs, as opposed to using an individual-based model representation for each mosquito. The ODE population level model is an upscaling, or homogenization, of the computationally more expensive models that track many individual mosquitoes. In effect, the model is a summary representation of the finer scale mosquito population dynamics, for which we often lack appropriately scaled demographic data necessary to model individual mosquitoes. The mosquito population in a patch depends upon the local environment, weather, and mitigation strategies, thus, the ODEs are based on the assumption that the mosquito dynamics take place on a much smaller scale than the individual dynamics of the host agents. The risk of being bitten by a mosquito depends upon the local mosquito density, host density, and type of host activities. The probability of disease transmission depends on the disease prevalence in the patch, the biting rate of the mosquitoes, and the susceptibility of the individuals (Figure 2).

We used this method to adapt an ABM for host movement on a network to vector-borne diseases. Simulations illustrate scenarios for which the heterogeneity of an ABM is important to understanding the risk of an outbreak, disease dynamics, and effective mitigation strategies. We show that with spatial heterogeneity in host and mosquito density, varying host movement rates between patches can produce different results than a standard ODE model or ODE multi-patch model. We are using this method to adapt a complex ABM, EpiSimS [30], to model mosquito-borne disease in the USA. It will also be important to develop techniques for analysis of this hybrid model framework, including uncertainty quantification, sensitivity analysis, and determining the basic and effective reproduction numbers. Once implemented, this method will expand the capabilities of ABMs for complex host movement and decision-making to include an important class of diseases.

After developing the overall structure of the network-patch model, we describe how the force of infection is computed and give an approximate formulation for the basic reproduction number for each patch. We then present a detailed example of the methodology and results from simulations that illustrate the importance of accounting for host movement and heterogeneity in mosquito habitat for understanding and controlling mosquito-borne disease.

## 2. The model

The patch and ABMs are propagated simultaneously and coupled through the mosquito biting rate and disease transmission. After describing the overall framework of the ABM host network and ODE mosquito models, we provide a detailed description of how the infection spreads between the two populations. In the next section, we present a simple ABM to illustrate the governing principles of the network-patch model.

### 2.1. The agent-based host model

We follow the general idea of the ODD protocol outlined in [20] to describe the agent-based (or individual-based) portion of the model. Host movement is defined by an ABM on a network of locations and the activity of each agent is tracked during their daily activities. The exact implementation of host movement will depend upon the ABM being used and on the questions asked. In general, however, the location of each host agent is updated at chosen discrete time intervals based on set movement and activity rules [8,17,19,30]. At any specific time, each agent or individual exhibits characteristics, such as their current activity, susceptibility to infection, infection status, and patch location, *k*. The agents move between connected location or activity nodes as determined by an underlying movement model. The activity patterns might come from a complex agent-based simulation, such as EpiSimS, or a simple random walk algorithm where the individuals randomly change their location/activity at the end of each time step. This method is designed to be flexible enough to use for different ABMs.

#### 2.1.1. Purpose, entities, state variables and scales of ABM

We use an ABM developed for host movement on a network where each node is a location. The purpose of the original ABM was to simulate human movement on a network in order to model directly transmitted infectious diseases such as influenza across various network connectivity and host movement regimes. The model depicts a certain number of agents representative of humans moving between a network of locations while spreading an infectious disease.

Each agent is assigned an initial node location. At each time step, individuals can either move or not move depending on the user-defined probability of movement. If the individual does decide to move, a node to move to is chosen. The model assumes that agents can only move directly to nodes with an edge connecting to the currently occupied node. The rate the mosquitoes become infected in a patch depends on the number of infected people at node locations in the patch. This in turn creates increased risk of infection for individuals based at nodes within the same mosquito patch. Once infected, an individual will progress to an exposed/incubating stage where they are infected but not yet infectious. Next, the exposed/incubating agent will move to the infectious (and usually symptomatic) stage and, finally, to a recovered/immune stage. The distribution of time spent in each of these stages is user defined.

#### 2.1.2. Process overview and scheduling

The model is initialized by randomly connecting the chosen number of locations/nodes with probability *p* using an Erdös-Rényi graph algorithm from NetworkX [21]. Initially, the agents are randomly assigned a movement rate (the average rate at which a person moves to a neighbouring location), a current disease state (set equal to susceptible for all but a few randomly selected infected individuals), and a randomly chosen initial location all chosen from user-defined distribution. Here, the movement, incubation (time in the exposed state), and recovery (time in the infectious state) rates are assumed to have a lognormal distribution (Table 2).

The agents are advanced in fixed increments, Δ*t*, in time. During a time step, the infection status of each individual is updated. Human movement within the model takes place at the end of the time step after the disease states have been updated. The movement rate *ρ* is chosen from a probability distribution *M* that determines the likelihood that an agent will change location at each time step. That is, an agent will moved to another node with probability *Pr* = 1 − e *^−Δtρ^*, where *ρ* is a random sample from the probability distribution given in Table 2. Thus, a higher movement rate indicates that an agent is more likely to move to another node. If an agent does move, then the node to which it moves is selected with uniform probability from its neighbours. The model can be tuned to any desired time scale. For this paper, we have chosen a time scale of days with the model updating every 6 hours (0.25 days).

### 2.2. The mosquito model

The location and size of mosquito patches overlayed on the ABM can be different for different mosquito species. Although this can be implemented at any scale, we anticipate patches on the order of a building or a group of buildings (e.g. city block) when modelling a city or local habitat patches for animals. As hosts enter and spend time in a patch, their infection risk depends on the probability of being bitten by an infectious mosquito and their individual susceptibility to infection. The number of times an individual is bitten depends upon the activity they are engaged in, the number of mosquitoes and other hosts in the patch, and other environmental factors, such as the temperature or time of day. Activities (or locations) are mapped to a relative mosquito exposure parameter, *α* ∈ [0, 1], that defines the relative risk of an agent being bitten by a mosquito given an underlying risk for mosquito bites in the patch and the agent's activity. For example, for humans, outdoor activities might have a much higher biting rate than an indoor activity, especially if the building has screens and air conditioning. In general, risk will also depend on the mosquito species and habitat being considered.

We assume that the mosquito population dynamics depend on the mosquito species, temperature, photoperiod, and rainfall. Rainfall, or for some species, paradoxically, a lack of rainfall, can lead to mosquito population increases, which can lead to increased biting intensities experienced by humans. Mosquitoes successfully feeding on blood can lead to more eggs being oviposited and an increase in immature stages of mosquitoes [18,35]. Temperature affects the mosquito maturation rate (egg, larva, pupae), the extrinsic incubation period (EIP) [6,38], and the mosquito life span [15,23]. Vegetation, sanitation, land use, and building density also influence the mosquito life cycle. Some mosquito species, including *Aedes aegypti*, can complete most or all of their life cycle inside a human dwelling, resulting in less dependence on weather and more dependence on human behaviour. Field studies, detailed ABMs for *Aedes aegypti* population dynamics (e.g. Skeeter Buster [24]), and differential equation mosquito-borne disease models help define the relevant ODE model parameters.

The adult female mosquito population is divided into three epidemiological classes, susceptible (*S*), exposed (*E*), and infectious (*I*). The female mosquitoes emerge into the susceptible class, *S_v_* (where the subscript *v* stands for vector), and are exposed to infection after biting an infected human. Mosquitoes infected with the pathogen but not yet able to infect a susceptible human host by biting are in the exposed/incubating class. This period is referred to as the EIP. Once infected, a mosquito moves from incubating to infectious at the rate *ν_v_* with average time spent in the incubating, *E_v_*, class being 1*/ν_v_*. The EIP can depend on temperature, strain of the pathogen, or mosquito species [38]. After the incubation period, the mosquito moves from the exposed class to the infectious class, *I_v_*. The mosquito remains infectious for life, with an average lifespan depending on species and environmental conditions.

Adult female mosquitoes die at a per capita rate *μ_v_*, where 1*/μ_v_* is the average lifespan of an adult female mosquito in the given patch. This death rate can depend on exogenous factors such temperature, food availability, and humidity. Mitigation strategies focusing on increasing adult death (adulticides) can be included here via reduction of the adult female mosquito lifespan or chosen mosquito carrying capacity. The total adult female mosquito population is represented by *N_v_* = *S_v_* + *E_v_ + R_v_*.

Each mosquito patch is characterized by the scalar parameters (Table 1) specific to that patch. Patches are chosen so that the mosquito dynamics can be approximated as being homogeneously mixed and uniformly spatially distributed within a patch. Each model parameter, and thus the mosquito dynamics output, can depend upon the associated habitat patch, denoted by a patch index *k*. For the full model, every variable and parameter will have a superscript *k* to denote the particular patch location. However, for simplicity of notation, we suppress the superscript here. Our mosquito dynamics model for a patch is described as a system of ODEs as follows:

(1a) $$\frac{dS_{v}}{dt}=h_{v}\left(N_{v},t\right)-\lambda_{v}\left(t\right)S_{v}-\mu_{v}S_{v},$$

(1b) $$\frac{dE_{v}}{dt}=\lambda_{v}\left(t\right)S_{v}-\nu_{v}E_{v}-\mu_{v}E_{v},$$

(1c) $$\frac{dI_{v}}{dt}=\nu_{v}E_{v}-\mu_{v}I_{v}.$$

The total number of adult female mosquitoes, *N_v_ = S_v_* + *E_v_* + *I_v_*, includes all mosquitoes in the patch. The average force of infection to mosquitoes, *λ_v_(t)* (rate of infection for each mosquito per unit time), in the patch is defined as the product of the average number of bites per mosquito, the probability that a bite is on an infectious host, and the probability of transmission per bite. The details of defining *λ_v_(t)* will be given in the next sections.

The adult female mosquito per capita emergence function, *h_v_(N_v_*, *t)*, can be constant or vary with time as a function of the weather, host availability, density dependence in larvae, and other factors. We define emergence as *h_v_(N_v_*, *t)* = *(ψ_v_* − *r_v_N_v_/K_v_)N_v_*, where *ψ_v_* is the natural per capita emergence rate of female mosquitoes in the absence of density dependence. This term depends on egg-laying rates, probability of hatching, surviving larvae and pupae stages, and successful emergence as adult females. *K_v_* is the carrying capacity of the mosquitoes in the patch and *r_v_* = *ψ_v_* − *μ_v_* is the intrinsic growth rate of mosquitoes in the absence of density dependence. We include density dependence in the emergence function *h_v_*, rather than the adult mosquito death rate, because density dependence has been shown to occur in the aquatic larvae stage. However, the emergence rate can be simplified to be density independent, if desired, or adapted to a particular mosquito species or situation as needed. The complexity and structure of the mosquito model chosen depends upon the system considered and questions being asked.

Summing Equations (1), and using the definition of *h_v_(N_v_*, *t)*, the total mosquito population in each patch is modelled by

(2) $$\frac{dN_{v}}{dt}=\left(\psi_{v}-\frac{r_{v}N_{v}}{K_{v}}\right)N_{v}-\mu_{v}N_{v}=r_{v}\left(1-\frac{N_{v}}{K_{v}}\right)N_{v}.$$

In addition to varying between patches, all parameters can depend on time, weather, mosquito species, or other factors. For example, the mosquito lifespan can depend on temperature, so that the mosquito per capita death rate *μ_v_* = *μ_v_(T)* depends on temperature *T*. This model can be adapted to include additional classes such as separate egg and larvae classes or several infectious classes for the mosquitoes. For some pathogens, vertical transmission in mosquitoes can be important. This model can also be adapted to include vertical transmission (see, e.g. [12] for Rift Valley fever). Vector control measures can be incorporated explicitly to the different life stages of the mosquito via the emergence rate, death rate, changing the number of mosquitoes, or patch carrying capacity. We find that the important time-dependent parameter variations are seasonal mosquito recruitment rate, seasonal biting rate, seasonal mortality rate, and a temperature-dependent seasonal EIP.

Our current model is deterministic, but stochastic variations in the environmental variables, mosquito populations, and infectious status can all be important, especially in the early stages of an emerging epidemic when there are few infected mosquitoes in a patch. In these situations, the model can be modified to include these effects using approaches such as the one described in [2] for stochastic disease models.

### 2.3. Biting and infection rates

The ABM and mosquito ODE models communicate with each other through the force of infection parameters. In each patch *k*, the force of infection from mosquitoes to hosts, $\lambda_{h,j}^{k}$ (where the subscript *h* refers to hosts and the subscript *j* refers to activity), is communicated to the ABM. This is used to determine the actual probability of being bitten by an infectious mosquito, based on each agent's activity and associated relative mosquito exposure modifier, *α_j_*. In the same way, the probability of a susceptible mosquito biting an infectious host in the patch is used to compute the mosquito force of infection, $\lambda_{v,j}^{k}\left(t\right)$, and is communicated back to the ODE equations (1) via the total number of agents, $N_{h}^{k}$, and the number of infectious agents, $I_{h}^{k}$, in each patch as well as the relative mosquito exposure of each agent, *α_j_*.

#### 2.3.1. Biting rate

The force of infection for the spread of the epidemic depends on the mosquito-host contact rate, or biting rate. Each location and activity in a patch will have an associated level of relative potential exposure to mosquitoes, *α_j_* with 0 ≤ *α_j_* ≤ 1. For example, if a location is outside, the potential for exposure to biting mosquitoes is high so we assume *α* = 1.0. For humans, a more moderate risk location could be a building with no screens, faulty screens, or no AC for which we may assume 0.5 ≤ *α* ≤ 1.0. Buildings with screens and/or AC are considered low risk so 0.0 ≤ *α* ≤ 0.5 is assumed. The assumptions will depend upon host species, mosquito species, and habitat. For example, mosquito populations which are ovipositing and hatching indoors may require different methods to calculate risk of exposure.

We define *σ_v_* as the total number of (successful) bites a single mosquito would have (per unit time) if hosts are plentiful. This can depend upon the gonotrophic cycle length (i.e. process of feeding on blood, resting, and egg laying), the weather, and mosquito species. The number of bites a host can sustain over a given time, *σ_h_*, depends on exposed skin area, attempts to deter biting (such as swatting and repellent), location (outside, inside with screens, inside with AC), and other mitigation strategies.

In a patch *k*, $\sigma_{v}^{k}N_{v}^{k}$ is the total number of bites that all the mosquitoes in a patch would like to make per unit time and $\sigma_{h}^{k}{\hat{N}}_{h}^{k}$ is the maximum number of bites available to the mosquitoes. The variable ${\hat{N}}_{h}^{k}$ represents the total number of hosts in patch *k*, scaled by their availability to be bitten. If all the hosts are at full risk of being bitten (*α* = 1), then ${\hat{N}}_{h}^{k}=N_{h}^{k}$, the total number of hosts in patch *k*. If there are people engaged in multiple activities in a patch with different risks of being bitten, then ${\hat{N}}_{h}^{k}=\sum_{j}\alpha_{j}N_{h,j}^{k}$. Here, $N_{h,j}^{k}$ is the number of people in patch *k* engaged in activity *j*.

We model the total number of contacts (successful bites) between hosts and mosquitoes in patch *k* with the function [10–12,26] as

(3) $$b^{k}=b\left(N_{v}^{k},{\hat{N}}_{h}^{k}\right)=\frac{\sigma_{v}N_{v}^{k}\sigma_{h}{\hat{N}}_{h}^{k}}{\sigma_{v}N_{v}^{k}+\sigma_{h}{\hat{N}}_{h}^{k}}.$$

This formula has the correct limiting behaviour as the number of mosquitoes or hosts approaches zero or infinity and has meaningful contact rates for any vector-to-host ratio. Other commonly used contact rates, such as frequency-dependent contact or density-dependent contact, do not have the correct limiting behaviour as populations vary greatly in time. However, if the vector-to-host ratios are known to stay within a certain range, then any of these contact formulations can be tuned to give approximately the same biting rates and *b^k^* can be changed to the desired contact formulation.

We define $b_{v}^{k}=b_{v}\left(N_{v}^{k},{\hat{N}}_{h}^{k}\right)=b^{k}∕N_{v}^{k}$ as the number of bites per mosquito per unit time in patch *k*. As the mosquito population gets low or the host population gets very large (i.e. the vector-to-host ratio is small), the number of bites is limited primarily by mosquito density. In this situation, the number of bites per mosquito is close to $\sigma_{v}^{k}$ and the number of bites per host is close to $\sigma_{v}^{k}N_{v}^{k}∕{\hat{N}}_{h}^{k}$. When the mosquito population is very large or the host population is low (i.e. the vector-to-host ratio is high), as can occur with pronounced seasonality, then the number of bites on hosts can be limited by the density of hosts. In this case, the number of bites on a host per mosquito is close to $\sigma_{h}^{k}{\hat{N}}_{h}^{k}∕N_{v}^{k}$ and the number of bites per host is close to $\sigma_{h}^{k}$ [10,11].

Similarly, $b_{h}^{k}=b_{h}\left(N_{v}^{k},{\hat{N}}_{h}^{k}\right)=b^{2}∕{\hat{N}}_{h}^{k}$ is the average number of bites per host per unit time and, although we assume that, on average, all the mosquitoes have the same biting rate, not all of the hosts are being bitten at the same rate. The average number of bites per host per unit time in patch *k* engaged in activity *j* per unit time is $b_{h,j}^{k}=\alpha_{j}b_{h}^{k}$, so that $\sum_{j}b_{h,j}^{k}N_{h,j}^{k}=\sum_{j}\alpha_{j}b_{h}^{k}N_{h,j}^{k}=b_{h}^{k}{\hat{N}}_{h}^{k}=b^{k}$ and the total number of bites in the patch are preserved.

#### 2.3.2. Infection rates

Susceptible mosquitoes are infected at a rate $\lambda_{v}^{k}\left(t\right)$ defined as the product of the number of bites one mosquito has per unit time, $b_{v}^{k}$; the probability of disease transmission (per bite) from an infectious host to the mosquito, *β_vh_*; and the average probability that the bitten host is infectious. The probability, *β_vh_*, of contracting the pathogen after biting an infectious host depends upon the infectiousness of the host and the susceptibility of the mosquito. If the bites in the patch are uniformly distributed among all the hosts in the patch, then the probability that the bitten host is infectious is *I_h_/N_h_*. However, if the probability of being bitten depends upon the activity the individual is engaged in, then the biting rate is not homogeneously distributed and the average probability that the bitten host is infectious is proportional to ${\hat{I}}_{h}^{k}∕{\hat{N}}_{h}^{k}$, where the scaled infected population in the patch is defined as ${\hat{I}}_{h}^{k}=\sum_{j}\alpha_{j}I_{h,j}^{k}$. Multiplying these three factors together, we have the infection rate for mosquitoes is

(4) $$\lambda_{v}^{k}=b_{v}^{k}\cdot \beta_{vh}\cdot \left(\frac{{\hat{I}}_{h}^{k}}{{\hat{N}}_{h}^{k}}\right).$$

This rate is used directly in model (1), thereby coupling the ABM and the mosquito ODE model.

The rate at which hosts are infected from infectious mosquitoes is the product of the number of bites a typical host engaged in activity *j* gets per unit time, the probability that a bite is from an infectious mosquito, and the probability of successful infection given a bite from an infectious mosquito. This rate can be expressed as

(5) $$\lambda_{h,j}^{k}\left(t\right)=\left(\alpha_{j}b_{h}^{k}\right)\beta_{hv}\left(\frac{I_{v}^{k}}{N_{v}^{k}}\right)=b_{h,j}^{k}\cdot \beta_{hv}\cdot \left(\frac{I_{v}^{k}}{N_{v}^{k}}\right).$$

This is approximately the risk, per unit-time, that a susceptible person engaged in activity *j* in patch *k* will be infected.

We advance the ABM using discrete time steps Δ*t*, and update the disease status and location of people using Markov Chain techniques. The infection rate $\lambda_{h,j}^{k}\left(t\right)$ must be converted into a probability, $p_{j}^{k}$, that a susceptible person in patch *k* engaged in activity *j* becomes infected in a time step. We assume the time to infection is exponentially distributed, so the probability of infection at the end of the time interval, Δ*t*, given that the individual was uninfected at the beginning of the time interval, is 1 minus the probability that the person is not infected, or

(6) $$p_{j}^{k}=1-e^{-\lambda_{h,j}^{l}\Delta t}.$$

This probability is used by the ABM to determine whether a susceptible agent becomes infected in a given time step. The transition equations, and probability of transition, for the state of an agent are

(7a) $$S_{h}\mapsto E_{h}:\mathrm{Pr}=1-e^{-\Delta t\lambda_{h,j}^{k}},$$

(7b) $$E_{h}\mapsto I_{h}:\mathrm{Pr}=1-e^{-\Delta t\nu_{h}},$$

(7c) $$I_{h}\mapsto R_{h}:\mathrm{Pr}=1-e^{-\Delta t\mu_{h}}.$$

If there are $S_{j}^{k}$ susceptible hosts in activity *j* in patch *k*, then on each time step we generate a random number *r* ∈ [0, 1] for each susceptible host and declare the host infected if $r<p_{j}^{k}$. This is a simple, albeit computationally inefficient, approach for infecting agents. In future work, we will describe more computationally efficient methods for updating an agent's infection status.

### 2.4. Reproduction number and analysis

Although we do not have an explicit formula for the coupled hybrid-patch network model, we can calculate the effective reproductive number for each patch. For the fully homogenized model, the basic reproduction number can be computed exactly via two quantities [12,27]. The first is the average number of hosts infected by one infectious mosquito introduced into a fully susceptible population, *R_hv_*, and the second is the average number of mosquitoes a single infectious host would infect if introduced into a full susceptible population, *R_vh_*. These quantities can then be multiplied together to form the type reproduction number, $R_{0}^{T}=R_{vh}\cdot R_{hv}$, which is the average number of hosts that would be infected (via mosquitoes) from one infected host introduced into a fully susceptible population. The basic reproduction number, or number of new cases in the next generation from one infected individual introduced in a fully susceptible population is $R_{0}=\sqrt{R_{vh}R_{hv}}$. For mosquito-borne disease, the next generation for an infected host is infected mosquitoes and vice versa. The type reproduction number can be written for this model formulation as $R_{0}^{T}={\left(R_{0}\right)}^{2}$.

The *effective* reproduction number measures the average number of secondary cases resulting from an infected individual introduced into the population at any time point during an epidemic. It accounts for reduced susceptibility of a population over time as individuals become immune or are vaccinated. When a disease has reached its endemic equilibrium, the effective reproduction number is equal to one. The effective reproduction number can be approximated by multiplying the basic reproduction number by the proportion of the population that is currently susceptible (i.e. not infected or immune). We can then get a rough estimate for the effective reproduction number from host to vector, $R_{vh}^{k}\left(t\right)$, and the effective reproduction number from vector to host, $R_{hv}^{k}\left(t\right)$, for the network-patch model at time *t* in patch *k* as

(8) $$R_{hv}^{k}\left(t\right)=\left(\frac{\nu_{v}}{\mu_{v}+\nu_{v}}\cdot \frac{\sigma_{v}}{\mu_{v}}\cdot \frac{\sigma_{h}{\hat{N}}_{h}^{k}}{\sigma_{h}{\hat{N}}_{h}^{k}+\sigma_{v}N_{v}^{k}}\cdot \beta_{hv}\right)\cdot \left(\frac{\left({\hat{N}}_{h}^{k}-{\hat{I}}_{h}^{k}\right)}{{\hat{N}}_{h}^{k}}\right),$$

(9) $$R_{vh}^{k}\left(t\right)=\left(\frac{\nu_{h}}{\mu_{h}+\nu_{h}}\cdot \frac{\sigma_{h}}{\mu_{h}+\gamma_{h}}\cdot \frac{\sigma_{v}N_{v}^{k}}{\sigma_{h}{\hat{N}}_{h}^{k}+\sigma_{v}N_{v}^{k}}\cdot \beta_{vh}\right)\cdot \left(\frac{S_{v}^{k}}{N_{v}^{k}}\right),$$

where the patch *k* superscript for the variables and parameters is suppressed. The terms ${\hat{N}}_{h}^{k}$, ${\hat{I}}_{h}^{k}$, $N_{v}^{k}$, and $S_{v}^{k}$ vary with time. However, the mosquito and disease parameters may vary with time as well. A full explanation for each of the non-dimensional terms in $R_{hv}^{k}\left(t\right)$ and $R_{vh}^{k}\left(t\right)$ can be found in [27]. Over short periods of time, the product $R_{hv}^{k}\left(t\right)\cdot R_{vh}^{k}\left(t\right)\approx R^{T,k}\left(t\right)$ will give us an estimate for the effective type reproduction number at time *t* in patch *k* for*R*the network patch model.

Methods for analysing and quantifying the results of ABMs for disease spread are still a relatively new area and there is much to be done in standardizing analysis of hybrid models combining ABMs with differential equations. The effective reproduction number heuristic shown here gives an estimate for the potential of disease spread given pathogen introduction, while the risk curve, *p^k^(t)*, shows actual risk of acquiring disease in patch *k* at that specific time in a particular run.

## 3. Network-patch example

We adapt an ABM for host movement and directly transmitted disease to a mosquito-borne disease in order to compare the effects of host movement and environmental heterogeneity on mosquito-borne disease spread. We describe the model coupling method and evaluate the model in the context of disease modelling by comparing output for a well-mixed baseline scenario to the corresponding standard ODE model for both humans and mosquitoes. We then illustrate how the network-patch model differs from the associated homogeneous mixing model, even when all the hosts have the same activity or exposure parameter.

Each location/node is assigned to patch of homogeneously distributed mosquitoes. We define the patch node density as the fraction of total nodes that are in a particular patch. We assume that all of the nodes have the same relative risk of mosquito exposure, meaning there is only one ‘activity’, *j* = 1, the associated exposure risk is *α_j_* = 1, and this risk depends only upon the mosquito population in the patch associated with each node. Therefore, the risk of a person being infected, or infecting mosquitoes, depends upon the average force of infection, $\lambda_{v}^{k}$ and $\lambda_{h}^{k}$, for the *k*th patch.

The network agent-based and mosquito differential equation patch models share information to calculate the force of infection as functions of the biting rate, the fraction of mosquitoes infected, and the scaled fraction of hosts infected in each patch. This minimal communication between the network and patch models allows the flexibility to easily adapt the approach to complex ABMs, such as EpiSimS [30]. The time step used in the ABM is also the amount of time that the mosquito patch model is progressed between communications. Thus, we feed the total number of agents, total number of infected agents, their associated exposure risks, and the time step being used to the mosquito patch model each time it is updated. The baseline risk of being bitten by an infectious mosquito is scaled in the patch by each agent or location's exposure risk. See Figure A5 for a flowchart of the ABM-patch model coupling method.

We tracked the proportion of agents infected in each patch for every time step and measured the contribution to overall infection burden of each patch. Finally, we tracked the risk, or $p_{j}^{k}=p^{k}$, in each patch as well as the effective and basic reproduction number. Note that the risk, $p_{j}^{k}$, depends on the time step chosen, mosquito habitat patch, relative density of mosquitoes and humans, and the proportion of mosquitoes in the patch that are currently infected.

### 3.1. Simulations

We consider a three-patch network as illustrated in Figure 1 for disease progression and mosquito parameters appropriate for dengue where the mosquito habitat and dynamics are determined by landscape, weather, available hosts, and breeding sites. We neglect mosquito movement between patches which is a reasonable assumption for *Aedes aegypti* mosquitoes, common vectors of dengue. We first establish a baseline case in order to compare different modelling assumptions as we incorporate heterogeneity. The model parameters (Table 1) for this baseline case are the same in each patch, and are constant in time. In the baseline case, each patch has the same density of mosquitoes and humans with the same resulting vector-to-host ratio. A high human movement rate was used for the baseline case to approximate a well-mixed human population. We simulated the baseline scenario 100 times to approximate the distribution of possible solutions created by the inherent stochasticity of the ABM. We solved the approximate mean-field ODE equations for the network-patch model using the mean values for human disease progression parameters from the ABM. The resulting ODE model for both humans and mosquitoes is the Manore *et al.* model [27] for dengue and chikungunya.

Figure A1 compares an ensemble of simulations for the baseline network-patch model with the ODE mean-field model. Although the distribution of solutions to the stochastic ABM are the same in each patch, we observe some differences in the samples. The mean-field ODE model for humans and mosquitoes is a good approximation of the stochastic network-patch model in this highly-mobile, well-mixed population with identical mosquito habitat patches. As we expected, our network-patch model matches the standard differential equation models well with some variation around the mean-field approximation. This allows for meaningful comparison of the hybrid model with the standard non-spatial ODE models that do not account for individual movement.

### 3.2. The impact of heterogeneity

Next, we included heterogeneity in patch parameters and in vector-to-host ratios as described in Figure 1. Patch 1 (green) is assumed to have locations including mostly buildings with AC and screens and not to have many mosquito breeding sites. Mosquitoes here have relatively low egg-laying and survival rates on average, so there is lower risk in this patch. Patch 2 (blue) has more human-made and/or natural breeding sites and fewer buildings with AC and screens. Thus, patch 2 has more humans at risk for mosquito bites. Mosquito populations in patch 2 are more robust than in patch 1 with medium density and an associated medium risk level. Patch 3 (red) is assumed to be prime mosquito habitat, having, on average, high mosquito density. Mosquito contact with humans in patch 3 is high, with locations consisting mostly of open air dwellings, buildings without screens, and outdoor locations. We assumed that patch 1 has the highest number of humans, patch 2 medium number of humans, and patch 3 the fewest humans.

The baseline version of the model for high human movement closely resembles results from a fully homogenized model with ODEs for both humans and mosquitoes (Figure A1). In this situation, justification for using the network-patch model rather than an ODE or stochastic differential equation model may be limited. However, this does provide a good baseline for comparison with other models and with the following scenarios that incorporate heterogeneity in patch parameters and host density and movement.

We considered three different host movement scenarios for heterogeneous habitat patches. Scenario 1 was high host movement between nodes, scenario 2 medium movement, scenario 3 low movement, and scenario 4 very low host movement (results not shown). Mosquito parameter values, human density, and mosquito density varied between the three patches. The highest human density and lowest mosquito density is in Patch 1 (green), medium human density and medium mosquito density in Patch 2 (blue) and lowest human density and highest mosquito density in Patch 3 (red). Thus, Patch 3 has the highest vector-to-host ratio and Patch 1 has the lowest vector-to-host ratio. We ran each of the scenarios 100 times to capture inherent stochasticity in the ABM. Stochasticity in placement of initial infectious humans and agent movement between patches is much more visible for the heterogeneous scenarios. Parameter values used for the runs can be found in Tables 2 and 3.

In the high host movement rate scenario, risk varies dramatically between patches. Since Patch 3 (red) had a high relative density of mosquitoes, it also had the highest patch risk after the epidemic became established (Figure A4, bottom row). Furthermore, more hosts became infected in Patch 3 than in any other patch (Figure A4, middle row). However, because of the high movement rates, if the population in each patch was sampled at a time point, one would not see higher prevalence in the highest risk patch. Therefore, it would be difficult to determine that the high risk patch should be targeted for mitigation in the presence of limited public health or mosquito control resources based on human prevalence data alone.

For the medium host movement rate scenario, agents are less likely to visit every patch often, so stochasticity in the initial location of infected agents and their subsequent movement has a greater effect on the overall dynamics (Figure A3). Also, once an agent is in a patch, it is more likely to stay there since the probability of moving is lower. Prevalence in the high risk patch is higher than in any other patch once the epidemic takes off. In this scenario, unlike the high movement scenario, the location of the high risk patch could generally be determined by prevalence data.

In the low (Figure A2) and very low (results not shown) host movement rate scenarios, we see much more heterogeneity between epidemics in the habitat patches. For example, the epidemic rarely peaks at the same time in the patches. This can be good from a control point of view, because once an epidemic is noticed in one patch, measures can be taken to reduce risk of spreading to the other patches. Prevalence is always higher in the high risk patch for the low movement scenario. Still, in the low movement case, the epidemic usually moves to every patch over the 200 days simulation time. For very low movement rates, the patches behave like separate villages with only sporadic connection between them. In the very low movement case, an ODE patch model with low stochastic movement rates between patches could probably capture the dynamics as well as the network-patch hybrid model. The network-patch model shows increasing sensitivity to the initially infected individual and their subsequent movement as patch heterogeneity increases and host movement rates are lower and more sporadic.

**Figure 3:**
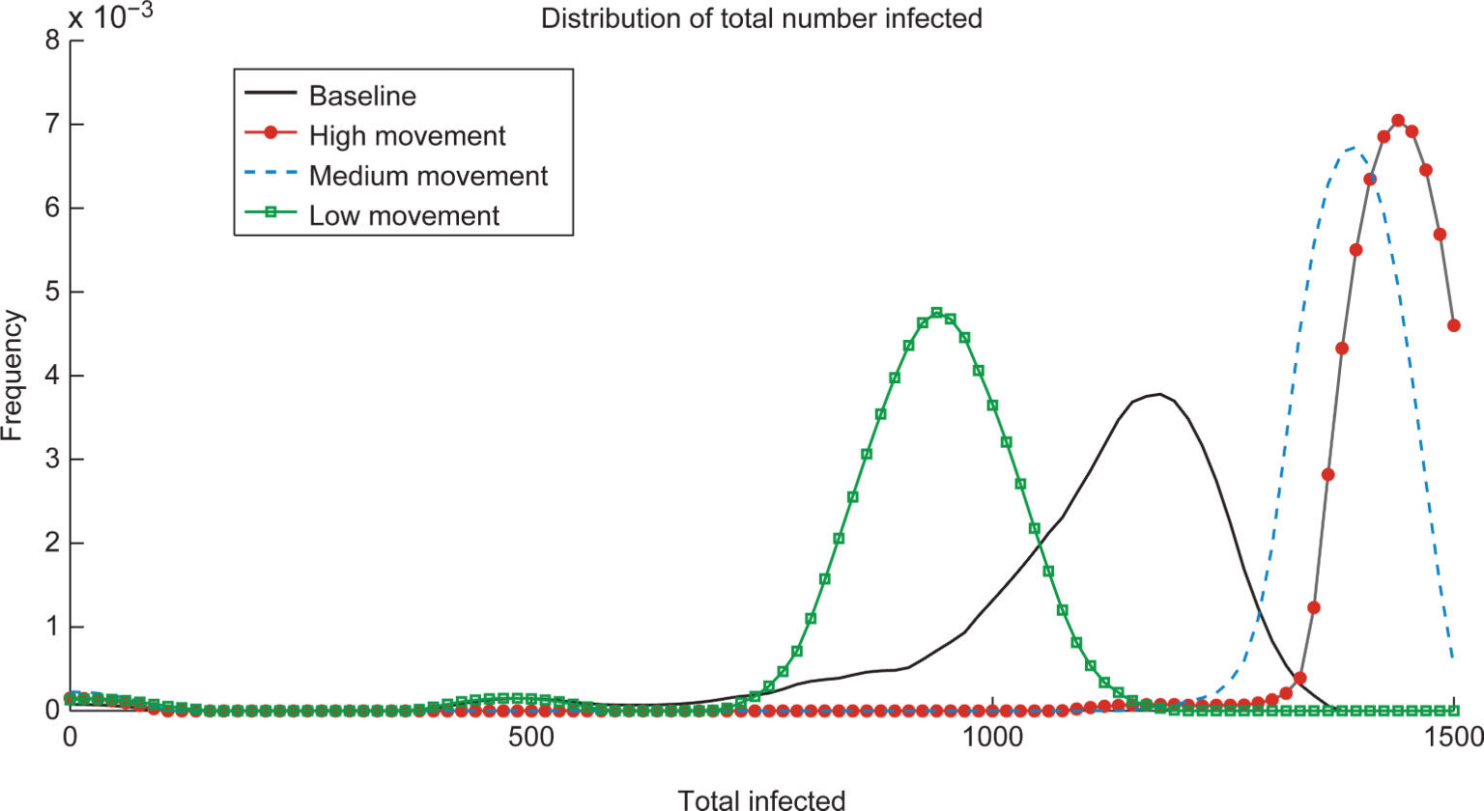
Distribution of the total number of people infected over the course of the simulation (200 days) for each scenario. Each scenario was run 100 times to capture intrinsic uncertainty due to stochasticity. The pathogen is introduced into a fully susceptible population of 1500 hosts with no mitigations implemented. In the heterogeneous scenarios with one high risk patch and high or medium human movement, the total consequence is higher than for the baseline homogeneous scenario. However, with low movement between patches, the scenario with one high risk patch results in lower total consequence than the baseline.

**Figure 4:**
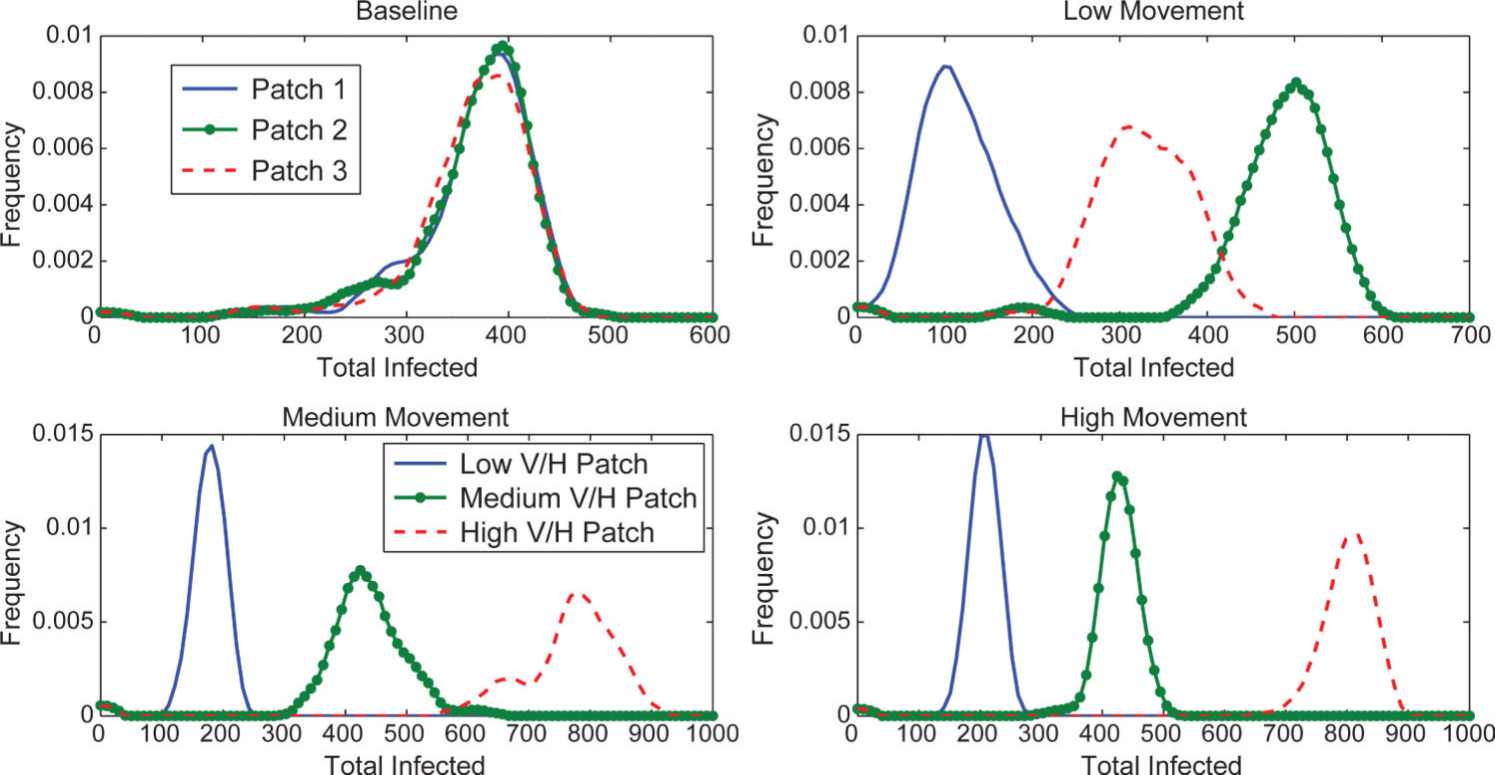
Distributions of the total number of hosts *initially infected* in each patch for the different scenarios. For the baseline case, all patches have the same density of mosquitoes and each patch is responsible for approximately the same number of initial infections. For the heterogeneous scenarios, red dashed is the high risk patch, green dotted the medium risk and blue solid the low risk patch. For high and medium host movement, the highest risk patch is responsible for the most infections. For the low movement scenario, the medium risk patch is responsible for the most infections and the low risk patch is responsible for the fewest. This difference from the high/medium movement scenarios can be explained by the fewer number of resident hosts in the high risk patch. Since movement between patches is low in the low movement scenario, the high risk patch runs out of susceptible hosts faster.

**Figure 5:**
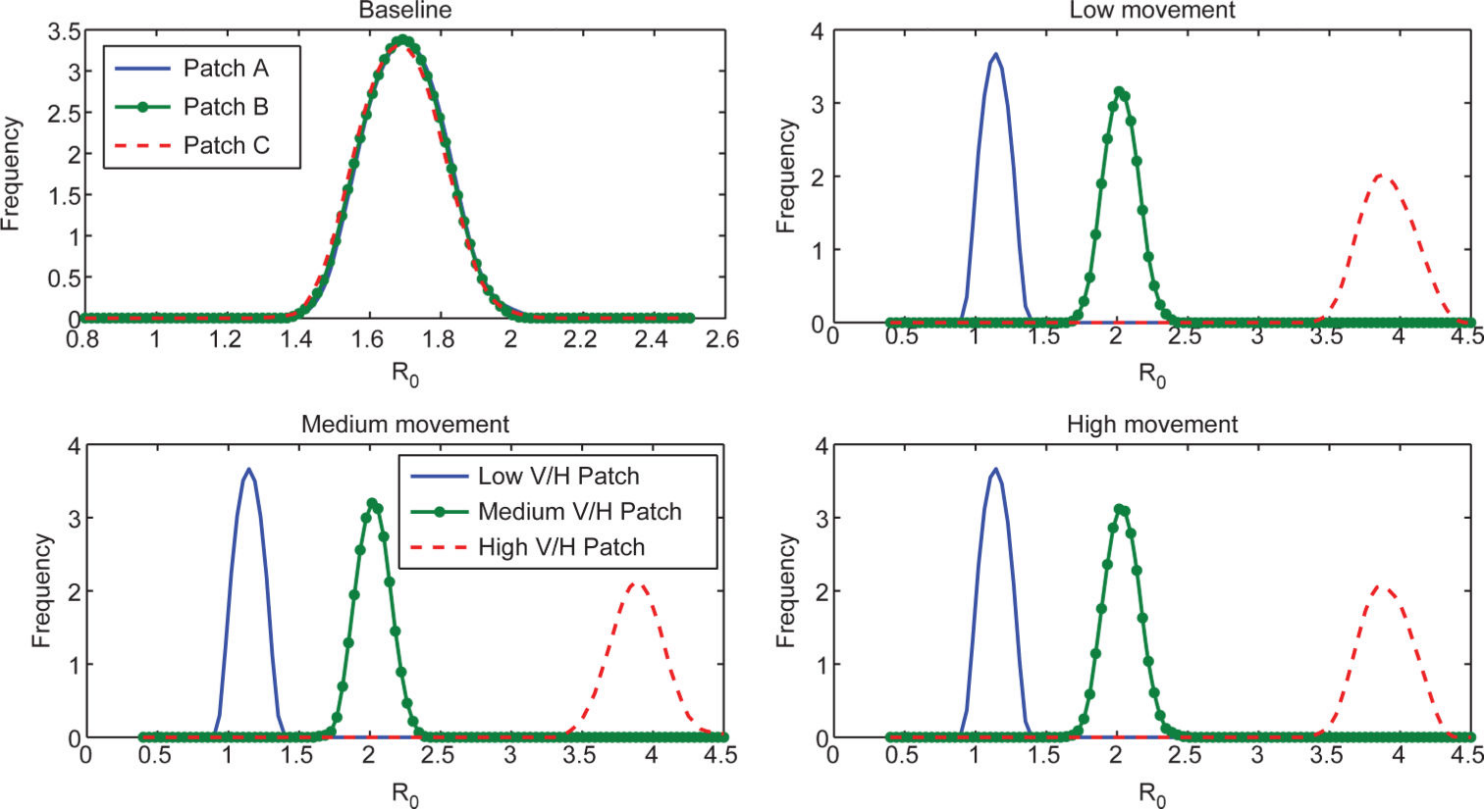
Distributions for the estimated basic reproduction number for each patch. For the baseline case, *R*_0_ ≈ 1.7 while in the heterogeneous cases, in the low risk patch *R*_0_ is just above 1, in the medium risk *R*_0_ is approximately 2 and in the high risk patch, *R*_0_ is just under 4. Notice that the basic reproduction number distribution (estimated as the effective reproduction number computed at the first time step for each run) is very similar among the heterogeneous scenarios. However, heterogeneity in movement patterns between patches results in different total consequence for each scenario as seen in Figure 4.

**Figure 6:**
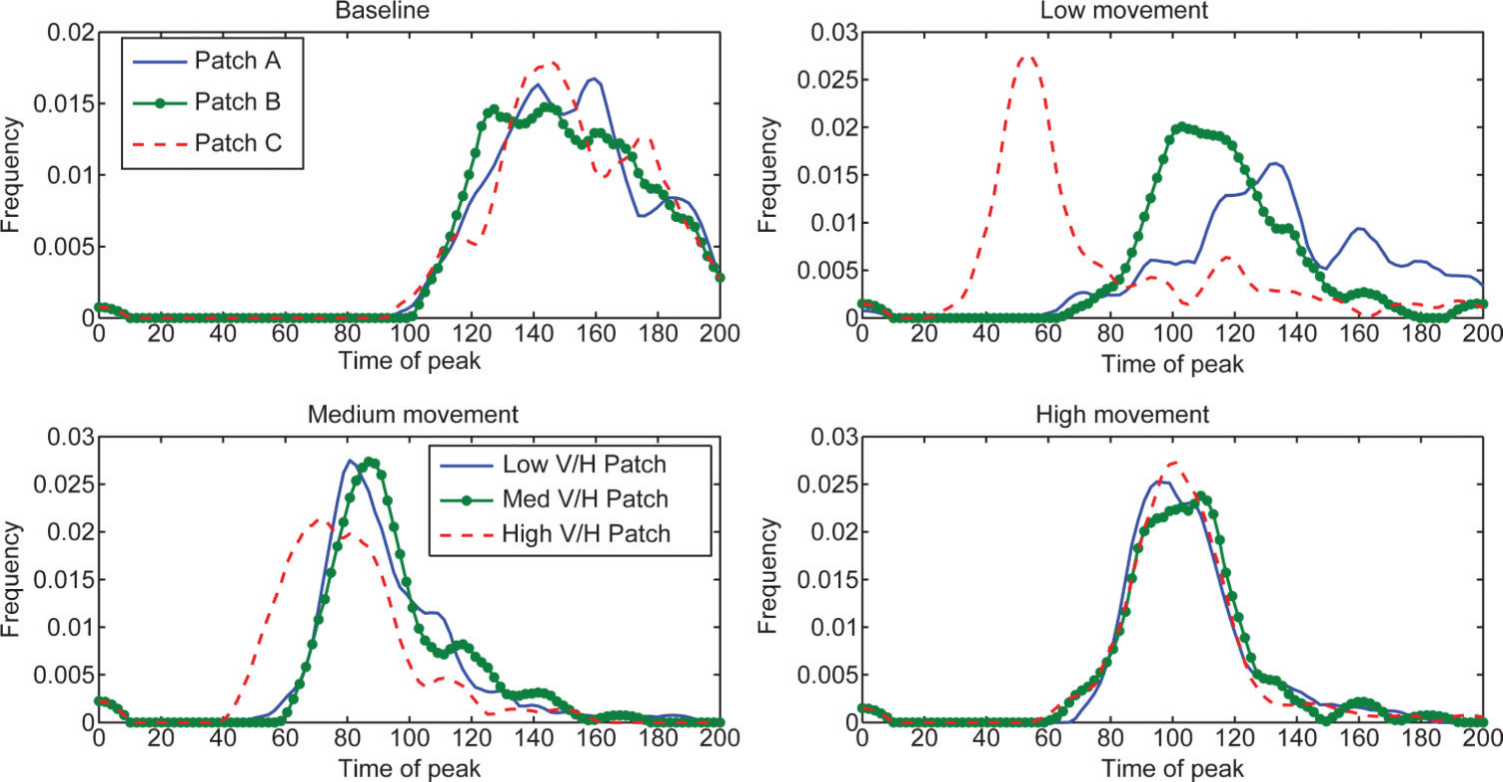
Distributions for the timing of the peak of the epidemic in each patch. For the low and medium movement scenarios, the high risk patch peaks before the other patches in general. For the high movement case and the baseline case, the patches reach the epidemic peak at approximately the same time. The low movement scenario has the most variation is epidemic timing.

**Figure 7:**
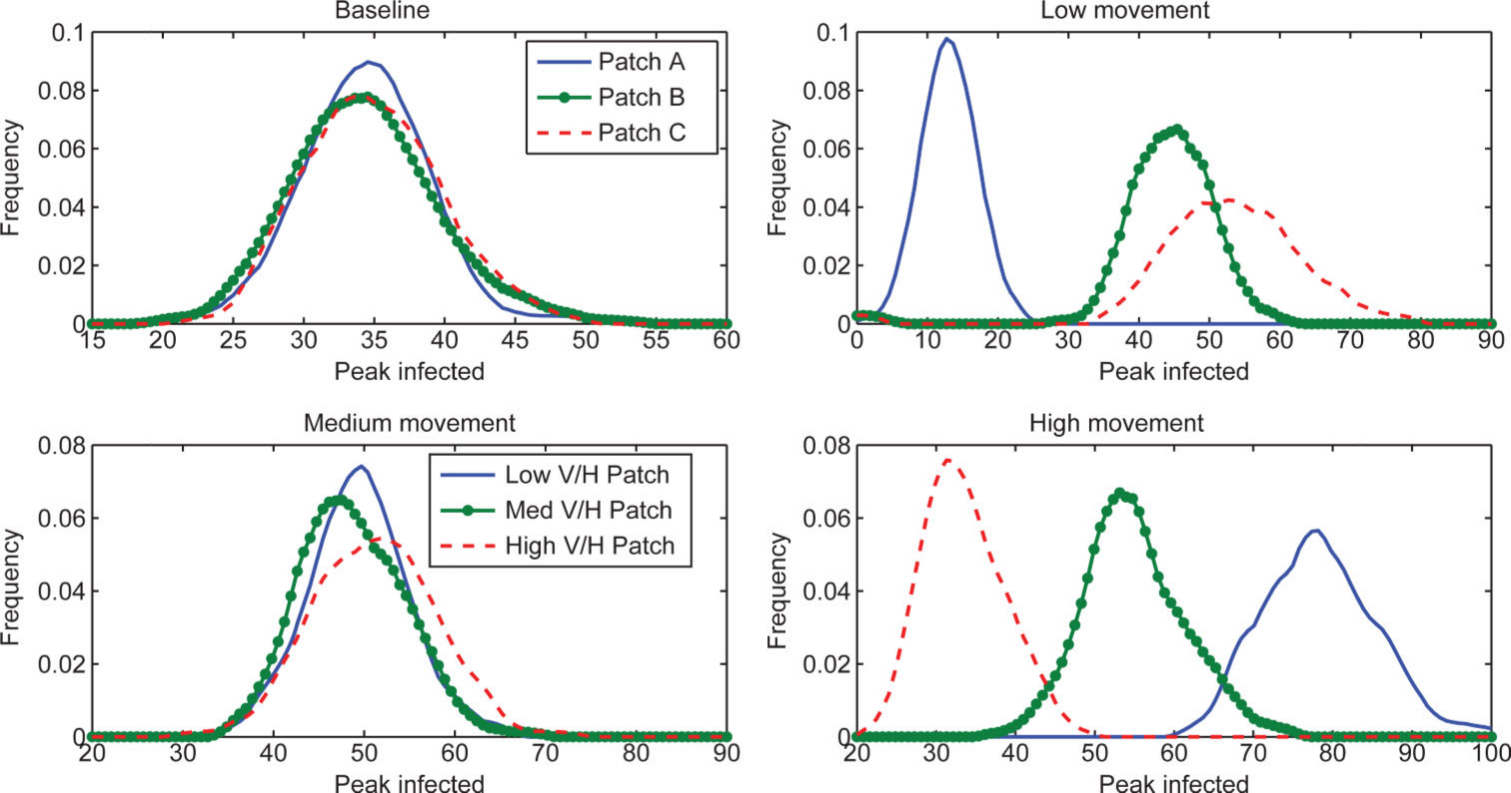
Distributions for the number of people who are infectious at the time of the epidemic peak in each patch. This is highly dependent on both patch risk and the movement patterns between the patches. The baseline and medium movement scenarios are the most similar for this metric, while the low and high movement rates have opposite patterns. This is again a reflection of the tradeoff between the risk of a patch from high vector density and the number of hosts in the patch and/or accessibility of the patch to hosts moving in and out.

The number of people infected is higher for the heterogeneous, high and medium human movement scenarios than for the baseline scenario (Figure 3) because of the presence of a high risk patch that is visited often by most agents due to the higher movement rates. We see that for the baseline scenario, each patch is responsible for initially infecting about the same number of hosts (Figure 4). However, for the heterogeneous scenario, the high risk patch is responsible for a relatively high number of initial infections, suggesting again that a targeted response may be effective. This is despite the fact that the distribution of the estimated basic reproduction number in each patch is the same for all movement scenarios (Figure 5). There is also more variation in timing of the epidemic peak across the runs for the heterogeneous scenarios (Figure 6). For the high movement scenario, there is enough host movement that the peak of the epidemic is at approximately the same time in all patches. However, for the low and medium movement scenarios, the epidemic tends to peak in the high risk patch first. The peak number of infectious people residing in each patch at the peak of the epidemic is shown in Figure 7 again highlighting the tradeoff between the risk of a patch from high vector density and the number of hosts in the patch and/or accessibility of the patch to hosts moving in and out. The figures illustrate that the network-patch model will capture heterogeneity that would not be captured by a differential equation model for humans and mosquitoes.

These simulation results show that heterogeneity in host movement and spatial heterogeneity in mosquito density can play an important role in the spread, timing and size of mosquito-borne pathogen epidemics. The network-patch model can capture this heterogeneity using the power of ABMs already tuned to particular host behaviour and landscapes. The importance of spatial and behavioural heterogeneity has also been observed for directly transmitted pathogens and in some current studies on mosquito-borne viruses such as dengue [7,13]. Capturing this inherent heterogeneity can be important for biosurveillance, mitigation, and treatment during outbreaks. This motivates the need for adapting ABMs with detailed host activity, behaviour, social, demographic, and geographical data to mosquito-borne diseases as a step towards creating real time and spatially explicit risk maps and mitigation strategies.

### 3.3. Mosquito heterogeneity across time and species

**Figure 8:**
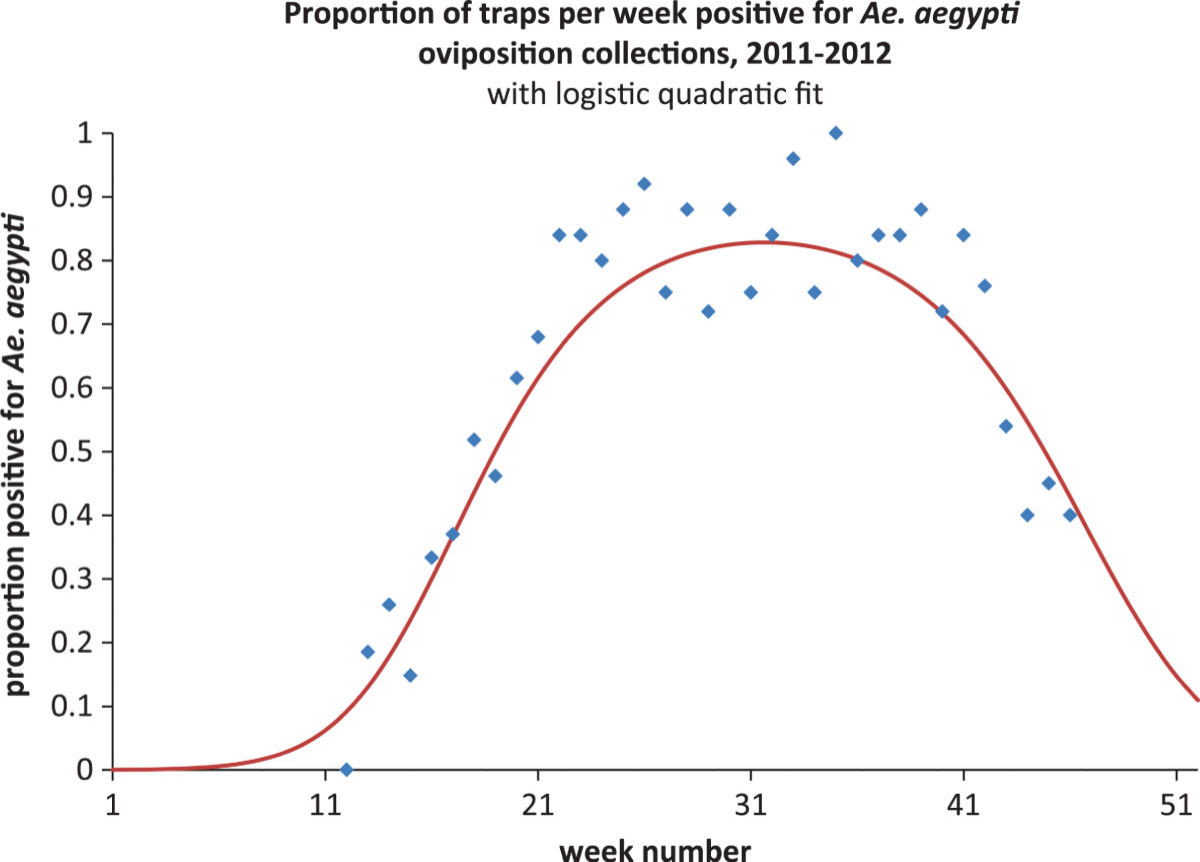
Example of seasonality in mosquito populations from New Orleans mosquito trap data [40]. Mosquito carrying capacities, emergence and/or death rates can be adjusted to follow seasonal patterns.

The illustrative example given above is for dengue spread in humans by one mosquito species, *A. aegypti*. We assumed constant density of mosquitoes in each patch across time. However, data about seasonal variation mosquito density such as in Figure 8 can be incorporated into the mosquito patch model by varying mosquito carrying capacities, $K_{v}^{k}\left(t\right)$, emergence rates, $\Phi_{v}^{k}\left(t\right)$, and/or death rates, $\mu_{v}^{k}\left(t\right)$ with time. Methods such as parameter fitting or data assimilation can be used to determine how the parameters change with time, temperature, policy, etc. Although we used humans movement to illustrate the method, an ABM for cattle movement between farms and/or wild animal movement between habitat could be adapted to consider Rift Valley fever infection. Bird movement in urban habitat could couple with the mosquito patch model to model West Nile virus risk.

The network-patch framework can also incorporate multiple mosquito species in the model. In a mixed mosquito species scenario, one patch can have *A. aegypti* mosquitoes while another has *A. albopictus* or some patches could contain both species. Including two mosquito species will be necessary to model emergence of chikungunya, which is more efficiently transmitted by *A. albopictus* than dengue is, thus necessitating inclusion of both *A. albopictus* and *A. aegypti*. The two species of mosquitoes differ in traits including human biting rates and vector competence which can be captured by adapting the parameter values for the mosquito model to each species. The network-patch model can incorporate overlap between the species by adding equations to the patch model for an additional mosquito species so that areas in the city can contain both species simultaneously. For Rift Valley fever or West Nile virus, both *Aedes* and *Culex* mosquitoes may be important and have very different dynamics and responses to climate and land use. This illustrates the ability of the model to incorporate multiple layers of mosquito habitat and populations to include different mosquito species and behaviour.

## 4. Discussion

We describe a method for coupling an ABM for directly transmitted diseases in hosts with a mosquito patch model in order to model mosquito-borne diseases while capturing important spatial, temporal, and behavioural heterogeneity. This approach makes use of the considerable infrastructure already available in large ABMs and expands the scenarios under which these models can be used. We used the model to explore mosquito-borne virus spread in heterogeneous environments illustrating the utility of the network-patch approach.

The examples show that heterogeneity in mosquito habitat and host movement can change the dynamics of the initial spread and spatial patterns of a mosquito-borne disease. We investigated the importance of heterogeneity in mosquito population dynamics and host movement on pathogen transmission, motivating the utility of detailed models of individual behaviour and observed that the random mixing model only captured the dynamics of the high movement rate scenario. Our hybrid agent-based/differential equation model can quantify the importance of the heterogeneity in predicting the spread and invasion of mosquito-borne pathogens. We observed that the total number of infected people is greater in heterogeneous patch models with one high risk patch and high or medium human movement than it would be in a random mixing homogeneous model. However, when there is low movement between patches, the scenario with one high risk patch resulted in lower total consequence than the baseline. Mitigation strategies can be more effective when guided by realistic models such as models outlined here that extend the capabilities of existing ABMs to include vector-borne diseases. Mitigation and prevention strategies can be optimized with better understanding of interactions between space, climate, and host movement resulting in observed heterogeneities. Future work will adapt ABMs such as the Epidemic Simulation System (EpiSimS) [17,29,37] and FRED [19] to model mosquito-borne diseases such as dengue and chikungunya in areas that are at risk.

The patch approach for creating dynamic risk maps by coupling host social network and movement models with the environment can be adapted for other scenarios such as environmental contaminants or other spatially and temporally varying hazards. Feasibility of using this approach for the specific phenomenon being modelled is determined by the temporal and spatial scales at which the host agents move and progress through the disease as well as the spatio-temporal scales that the environmental hazards change over, whether the environmental hazard is mosquitoes, livestock, birds, other wildlife, etc.

In applying this framework one should keep in mind that the ODE model of risk represents a homogenization of a large number of random events. This is applicable in mosquito-transmitted diseases where the vectors can be approximated as risk ‘clouds’ at some scale, but may be less applicable in other situations. The underlying patch model for the hazard being modelled could be of many different forms, including discrete, statistical, or Markov chain models, provided it can communicate an appropriate risk to the ABM. The mosquito population dynamics and disease ODE model can be adapted to multiple pathogens. However, the network-patch approach for coupling a mosquito model to an ABM described in this paper would work well for most standard homogeneous mosquito-borne disease models.

The hybrid network-patch model can be adapted to other mosquito-borne pathogens such as dengue and chikungunya, as well as pathogens spread by other arthropod vectors, such as lyme disease, with an appropriate underlying model for ticks. This method could also be adapted to zoonotic mosquito-borne diseases such as Rift Valley fever or West Nile virus and could be coupled with a spatial model for livestock and/or wildlife. If movement and heterogeneity among individual animals is important, the additional animal hosts could be incorporated as agents that would couple to the mosquito model or, alternatively, incorporated as another layer of patch ODEs. For example, a model for West Nile virus could be implemented by layering patches of bird habitat over a city and modelling bird dynamics and transmission by systems of differential equations that are coupled with the mosquito patches. Movement of birds (or mosquitoes) between patches could be added if needed. Parameters of a patch could change with time as weather or other factors affecting patch dynamics change, allowing for exploration of scenarios such as climate change. Patch-specific mitigation strategies as well as social strategies when agents avoid a patch with high hazard rates can also be implemented.

In conclusion, coupling ABMs for hosts with the environment extends the capability of existing tools to explore the role that spatial heterogeneity and host movement play in the emergence and spread of infectious disease, particularly mosquito-borne pathogens. We have presented a prototype for creating a dynamic risk map that changes with time as mosquito dynamics and host behaviour and movement change. In future work we will use methods arising in sensitivity analysis and uncertainty quantification to determine the most important factors in disease spread as indicated by these models. This approach can provide valuable insight into methods for disease control and lend important validation for simulation techniques.

## Supplementary Material

supplemental material

## Figures and Tables

**Table 1:** Parameters for the mosquito patch model and their dimensions. The range of parameter values and references are given in a table with the numerical simulations.

*ψ_v_*:	Per capita emergence rate of adult female mosquitoes (Time^–1^).
*μ_v_*:	Per capita death rate of adult female mosquitoes (Time^–1^).
*K_v_*:	Maximum number ofmosquitoes in the patch (Mosquitoes).
*σ_v_*:	Number of times one mosquito would want to bite a host per unit time, if hosts were freely available. This is a function of the mosquito's gonotrophic cycle (the amount of time a mosquito requires to produce eggs) (Time^–1^ ).
*σ_h_*:	The maximum number of mosquito bites an average host can sustain per unit time. This is a function of the host's exposed surface area, the efforts it takes to prevent mosquito bites, and any vector control interventions in place to kill mosquitoes or prevent bites (Time^–1^).
*β_hv_*:	Probability of transmission of infection from an infectious mosquito to a susceptible host given that a contact between the two occurs (Dimensionless).
*β_vh_*:	Probability of transmission of infection from an infectious host to a susceptible mosquito given that a contact between the two occurs (Dimensionless).
*ν_v_*:	Per capita rate of progression of mosquitoes from the exposed state to the infectious state. 1/*ν_v_* is the average duration of the latent period (Time^–1^).

**Table 2:** *Patch parameters*: the parameter values for the simulations experiments in Figures 3-7.

Parameter	Value (P1, P2, P3)	Explanation
*Baseline*		
*σ_h_*	(19, 19, 19)	Maximum bites on a human per day
*K_v_*	(1000, 1000, 1000)	Mosquito carrying capacity
Patch density	$\left(\frac{1}{3},\frac{1}{3},\frac{1}{3}\right)$	Fraction of locations per patch
Movement rate	$\ln\;N\left(\mu ,\sigma^{2}\right)$, mean = 1, var. = 0.001	Average number oflocation changes per day
*Heterogeneous patch, high movement*		
*σ_h_*	(5, 19, 30)	Maximum bites on a human per day
*K_v_*	(750, 1500, 3750)	Mosquito carrying capacity
Patch density	$\left(\frac{1}{2},\frac{1}{3},\frac{1}{6}\right)$	Fraction of locations per patch
Movement rate	$\ln\;N\left(\mu ,\sigma^{2}\right)$, mean = 1, var. = 0.001	Average number of location changes per day
*Heterogeneous patch, medium movement*		
*σ_h_*	(5, 19, 30)	Maximum bites on a human per day
*K_v_*	(750, 1500, 3750)	Mosquito carrying capacity
Patch density	$\left(\frac{1}{2},\frac{1}{3},\frac{1}{6}\right)$	Fraction of locations per patch
Movement rate	$\ln\;N\left(\mu ,\sigma^{2}\right)$, mean = 0.1, var. = 0.001	Average number of location changes per day
*Heterogeneous patch, low movement*		
*σ_h_*	(5, 19, 30)	Maximum bites on a human per day
*K_v_*	(750, 1500, 3750)	Mosquito carrying capacity
Patch density	$\left(\frac{1}{2},\frac{1}{3},\frac{1}{6}\right)$	Fraction of locations per patch
Movement rate	$\ln\;N\left(\mu ,\sigma^{2}\right)$, mean = 0.01, var. = 0.001	Average number of location changes per day
*Heterogeneous patch, very low movement*		
*σ_h_*	(5, 19, 30)	Maximum bites on a human per day
*K_v_*	(750, 1500, 3750)	Mosquito carrying capacity
Patch density	$\left(\frac{1}{2},\frac{1}{3},\frac{1}{6}\right)$	Fraction of locations per patch
Movement rate	$\ln\;N\left(\mu ,\sigma^{2}\right)$, mean = 0.001, var. = 0.001	Average number of location changes per day

Notice, for the simulations in the heterogeneous scenarios only the movement rate changes.

**Table 3:** *Patch parameters*: the parameter values used for all numerical experiments.

*All Experiments*		
Parameter	Value (P1, P2, P3)	Explanation
*ψ_v_*	(0.3, 0.3, 0.3)	Emergence rate of female mosquitoes
*σ_v_*	(0.5, 0.5, 0.5)	Max mosquito bite demand per day
*β_hv_*	(0.33, 0.33, 0.33)	Probability of M-to-H transmission
*β_vh_*	(0.33, 0.33, 0.33)	Probability of H-to-M transmission
*ν_v_*	(0.1, 0.1, 0.1)	Mosquito E-to-I rate
*μ_v_*	$\left(\frac{1}{14},\frac{1}{14},\frac{1}{14}\right)$	Mosquito death rate
*r_v_*	*ψ_v_* – *μ_v_* all patches	Intrinsic growth rate
*Patch-independent parameters*		
Total number oflocations	300	Distributed among patches by density
Edge probability	0.03	Prob. two locations connect
Total human pop.	1500	Distributed equally among locations
Initial infected %	0.5%	% initially infected per patch
Recovery rate	$\ln\;N\left(\mu ,\sigma^{2}\right)$, $\mathrm{mean}=\frac{1}{6}$, var. = 0.001	Avg. human recovery of 6 days
Incubation rate	$\ln\;N\left(\mu ,\sigma^{2}\right)$, $\mathrm{mean}=\frac{1}{5}$, var. = 0.001	Avg. human E-to-I of 5 days
Total simulation time	200 days	
ABM time step	0.25 days	
Mosquito r-k time step	0.005 days	
