# Supplementary material for: A network-patch methodology for adapting agent-based models for directly transmitted disease to mosquito-borne disease
Source: J Biol Dyn. Author manuscript; Available in PMC 2017 Jun 16. (PMC5473441; doi:10.1080/17513758.2015.1005698)
Supplement: supplemental material [file NIHMS667926-supplement-supplemental_material.pdf]

Appendix A. Supporting material

We present here three representative runs for each of the network-patch scenarios as well as a flow chart illustrating the modeling coupling process.

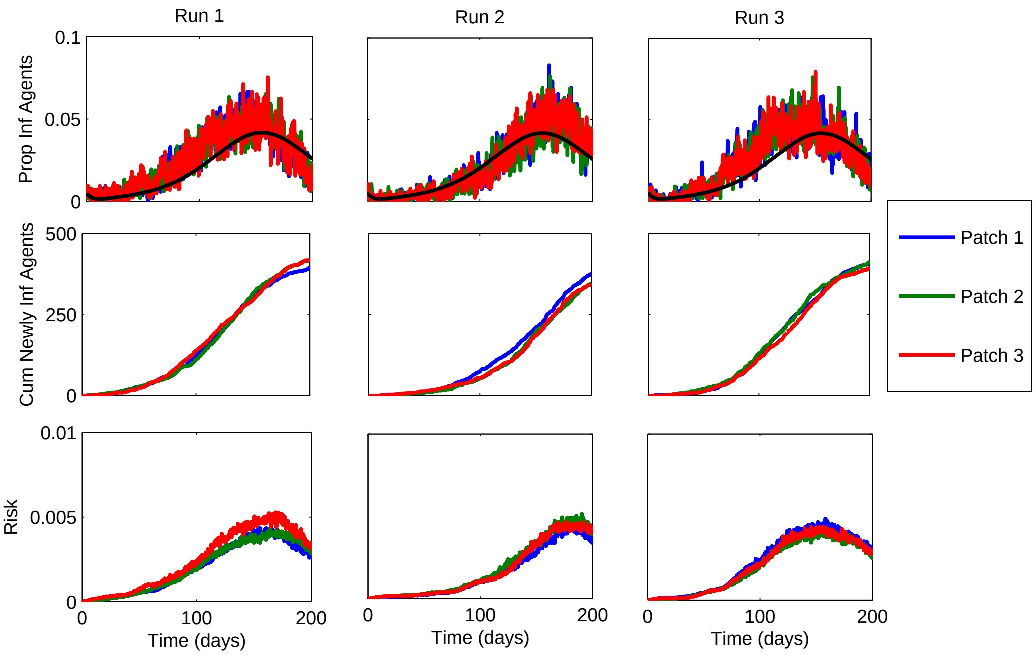

Figure A1. Three representative runs for the baseline scenario with the cumulative number initially infected in each patch and the risk in each patch over time. The risk can be thought of as the probability of an agent who was in the patch for 6 hours (for our simulations $\Delta t = 0.25$ days) becoming infected during that time. The ODE model solution is represented by a solid black line. The baseline case matches well with the well-mixed ODE model.

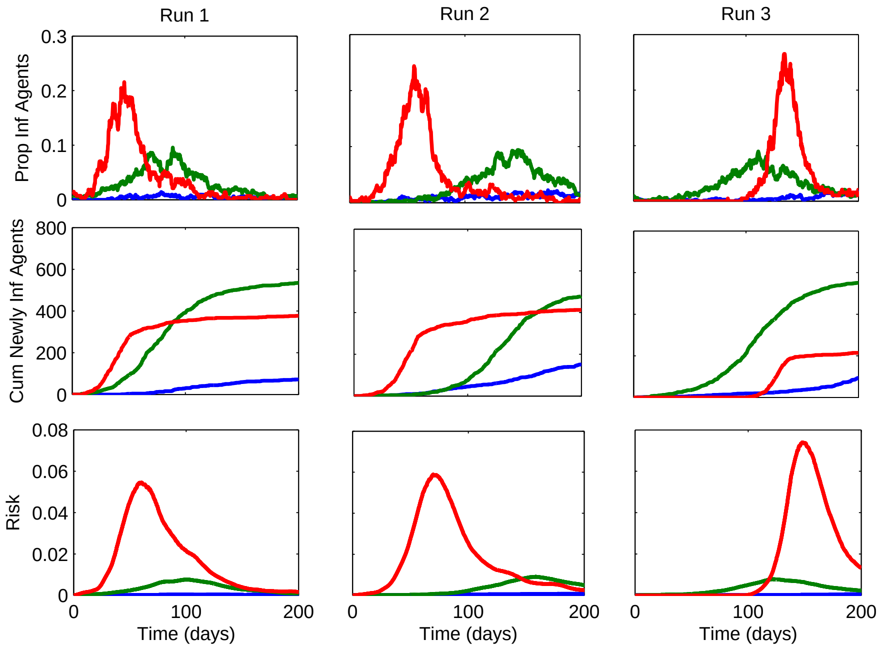

Figure A2. Three representative runs for the heterogeneous, low movement scenario with the cumulative number initially infected in each patch and the risk in each patch over time.

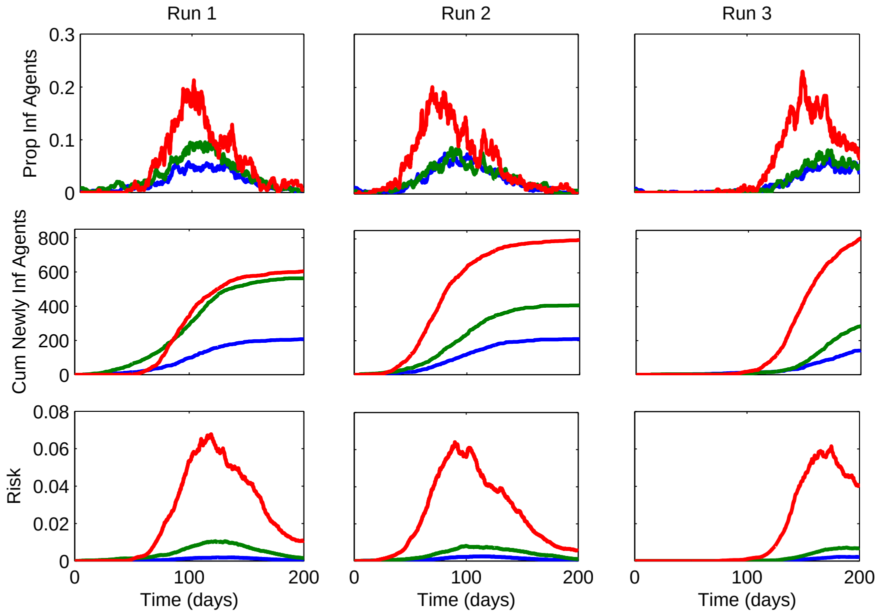

Figure A3. Three representative runs for the heterogeneous, medium movement scenario with the cumulative number initially infected in each patch and the risk in each patch over time.

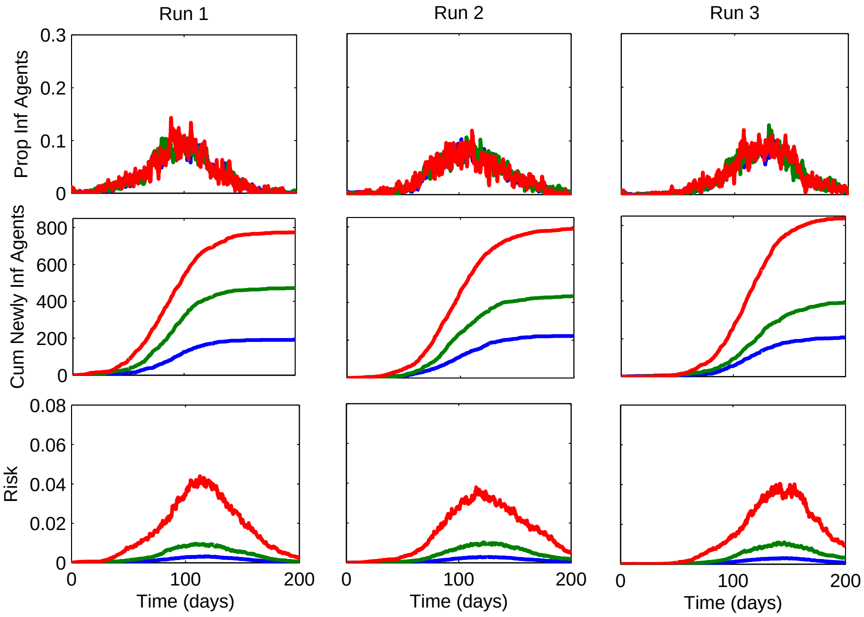

Figure A4. Three representative runs for the heterogeneous, high movement scenario with the cumulative number initially infected in each patch and the risk in each patch over time.

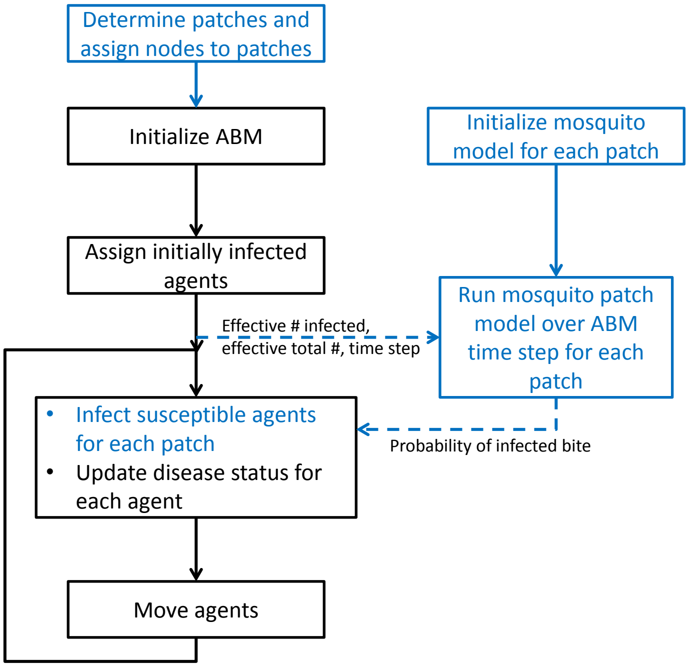

Figure A5. Flow chart for the ABM and associated patch model. Blue represents portions of the model that are added as a result of the network-patch approach. Dotted lines represent communication between the ABM and the patch model.
